# Effects of Doxycycline on Cx43 Distribution and Cardiac Arrhythmia Susceptibility of Rats after Myocardial Infarction

**Published:** 2014

**Authors:** Xi-zhen Fana, Hong-jun Zhu, Xu Wu, Ji Yan, Jian Xu, De-guo Wang

**Affiliations:** a *Department of Cardiology, Anhui Provincial Hospital, 17 Lujiang RoadLuyang District, Hefei, Anhui Province, 230001, P.R. China.*; b *University of Science and Technology of China, JinZhai Road Baohe District, Hefei, Anhui Province, 230026, P. R. China.*; c *Department of Cardiology, Yijishan Hospital, Wannan Medical College.*

**Keywords:** Doxycycline, Cx43, Cardiac arrhythmia, Matrix metalloproteinases, Myocardial infarction

## Abstract

This study aims to observe the effects of doxycycline (DOX) on gap junction remodeling after MI and the susceptibility of rats to cardiac arrhythmia. The proximal left anterior descending coronary artery of rats was ligated to establish a myocardial infarction animal model. DOX, methylprednisolone (MP), or vehicle was intraperitoneally injected into the animals for two weeks. Then, the heart size and heart function of all animals were determined through echocardiography. The experimental animals were sacrificed after the electrophysiologic study. Myocardial tissues were sampled to analyze the distribution of Cx43 using immunofluorescence; the Cx43 content was analyzed using western blot analysis; and the MMP-2 and MMP-9 activity in the myocardium was analyzed using gelatin zymography. The distribution of Cx43 in the border of the infarcted myocardia in the MI and MP groups was clearly disrupted and the Cx43 content was significantly reduced. In addition, the distribution of Cx43 in the border of the infarct in the DOX group was relatively regular, whereas two weeks of DOX treatment significantly inhibited MMP activity. Meanwhile, the induction rate of arrhythmia in the rats after DOX treatment was lower than those in the MI and MP groups. The results show that DOX treatment after myocardial infarction improves gap junction remodeling in the myocardial tissue near the infarcted area by inhibiting MMP activity and reducing susceptibility to cardiac arrhythmia.

## Introduction

Cardiac arrest is still the leading cause of death in developed countries. About 350000 people die of sudden cardiac death annually in the US, among which, about 2/3 of patients had previous myocardial infarction (MI). The vast majority of cardiac arrests are caused by arrhythmia, most of which is related to the border-zone of myocardial infarction (1). Previous studies showed that gap junction remodeling in the border of the infarcted area, which is mainly expressed as a Cx43 distribution disorder, is the key cause of myocardial conduction delay in the border of the infarct (2, 3). However, the reason for the disorder in Cx43 distribution in the border of the infarct area is still unclear. The expression and distribution of Cx43 are closely related to several conjunctional proteins, *e.g*., N-cadherin and vinculin, among others, build cell–cell connections or extracellular connections (4, 5). However, these junctional proteins may be hydrolyzed, and the intercellular junctions between the myocardial cells and extracellular matrix are impaired, accompanied by Cx43 redistribution, which are ascribed to the increased in MMP expression in the myocardial tissues after myocardial infarction. 

The increased MMP activity after myocardial infarction is closely related to the remodeling of the infarcted left ventricular and the deterioration of heart function. The MMP activation and extracellular degradation may result in left ventricular enlargement and heart failure (6). MMPs hydrolyze the matrix proteins of myocardial cells may impair cell–cell connections (7), MMPs can even hydrolyze N-cadherin directly (8). Our previous studies showed that N-cadherin plays a very important role in maintaining gap junctions and their function (9). Therefore, the occurrence of ventricular arrhythmia after myocardial infarction is related to the destruction of the connections between myocardial cells by MMPs, which affects the number and distribution of Cx43 and lower the electrical conductivity of gap junctions. Based on our hypothesis, suppression of MMP activity may attenuate the gap junction remodeling in the border of infarcted area and reduce the risk of ventricular arrhythmias post MI.

To study the effects of MMP suppression on gap junction remodeling and arrhythmias, we adopted an inhibitor of nonselective matrix metalloproteinases*, i.e*., doxycycline (DOX), to inhibit MMP activity. DOX, (4S, 4aR, 5S, 5aR, 6R, 12aS) – 4 - (Dimethylamino) – 1, 4, 4a, 5, 5a, 6, 11, 12a – octahydro – 3, 5, 10, 12, 12a – pentahydroxy – 6 – methyl – 1, 11 – dioxo – 2 - naphthacenecarboxamide hydrochloride, is a tetracycline antibiotic with an enlarged application range because of its nonselective MMP inhibition. In fact, dental surgery uses DOX to treat periodontal disease because of its inhibitory effect on MMP activity (10). Other tetracycline antibiotics have also been used as protective agents for the myocardium during short-term ischemia–reperfusion (11, 12). Previous literature reported that DOX improves the effects of left ventricular remodeling after myocardial infarction (13). The effects of DOX on the occurrence of arrhythmias after myocardial infarction remained to be clarified. Our research mainly aims to compare DOX with methylprednisolone (MP), which enhances MMP activity and inhibits inflammatory reactions, and to observe 1) the effect of DOX on MMP activity in the myocardium after myocardial infarction and its effect on the distribution of Cx43, and 2) the effect of DOX on the susceptibility of rats to arrhythmia in a myocardial infarction model.

## Experimental

*Preparation for surgery and drug treatment*

Male Sprague–Dawley rats (200 g to 250 g) were intraperitoneally anesthetized with pentobarbital (70 mg/Kg), intubated, and connected to a respirator. The heart was exposed using an angular incision on the left side of the thoracic cavity. The pericardium was opened to ligate the left anterior descending branch of the coronary artery. Then, the thoracic cavity was closed. After myocardial infarction, the experimental animals were divided into three groups: one group received DOX (25 mg/Kg/day, dissolved in deionized water) twice daily via intraperitoneal injection; one group received MP (50 mg/Kg/day) once a day via intraperitoneal injection and one group were fed a placebo twice daily (the deionized water was the same quantity as that of DOX). The dosages of DOX and of MP are the quantities that affect left ventricular myocardial infarction as reported in the literature (14). In addition, the sham operation group was subjected to thoracotomy using the same procedures above without ligation of the coronary artery.

*Study of cardiac electrophysiology*

Two weeks after surgery, the rats were anesthetized again, intubated, and connected to a respirator. The chest wall was opened to expose the heart. A customized bipolar electrode was placed on the apex of the right ventricle and attached to the epicardium for pacing. S1S2 stimulation with 130 ms of basic RR interval was used to determine the effective refractory period. Premature stimuli were started from 80 ms and were reduced by 5 ms each time until 20 ms. the burst stimulus (18S1s) was adopted when ventricular tachycardia was not triggered by the S1S2 stimulus. The stimulus interval was steadily increased from 20 ms, ascending by 5 ms each time until a 1:1 capture was formed. Each process was repeated three times. The occurrence of a ventricular tachycardia was monitored continuously during the experiment. A VT duration of >3 s was defined as sustained ventricular tachycardia. Statistical data was collected by replaying video. However, the observers blinded to the grouping of the subjects. 

*Morphological and functional analysis of the rat heart through* *echocardiography*

Echocardiography study were performed on six animals from each group (n=6) before the electrophysiology study. Two testers blinded to the test grouping conducted the study using an HP5500 cardiac ultrasound machine. The sampling line obtained an M-type ultrasound image passing through the right ventricular anterior wall, the nose of the bicuspid valve, and the posterior left ventricular wall after a 15 MHz probe was used to acquire the typical left ventricular long axis view in two dimensions. The diastolic and systolic left ventricular internal diameters were measured. As a measure of left ventricular (LV) function, the EF (ejection fraction) and fractional shortening (FS) were calculated.

*Fluorescence analysis of MMP-2 and MMP-9 activity MMP Zymography*

The hearts of the rats were removed after the electrophysiology study. Proteins were extracted from the frozen ventricle (n=6) after homogenization (16,17). Briefly, the homogenates were resuspended in lysis buffer, centrifuged at 12000_×*g*, and protein concentration was determined using the Bradford technique. The samples were diluted in sample buffer [125 mM Tris–HCl (pH 6.8), 20% glycerol, 4% SDS, and 0.04% bromophenol blue] and aliquots with a final protein content of 5 mg were separated by 10% SDS-polyacrylamide electrophoresis (SDS-PAGE) on gels containing 0.6% gelatin. After SDS-PAGE, the gels were washed twice in extraction buffer [20 mM Tris–HCl (pH 7.5), 2 mM CaCl_2_, 1 mM ZnCl_2_, 0.02% NaN_3_, and 2.5% Triton X-100] for 30 min each time and rinsed in water. The gels were incubated overnight at 37 °C in incubation buffer [20 mM Tris–HCl (pH 7.5), 2 mM CaCl_2_, and 1 mM ZnCl_2_], stained with Coomassie Blue staining solution (0.5% Coomassie R250, 30% methanol, and10% acetic acid) for 2 h, and then destained in distilled deionized H_2_O. Imaging software was used to analyze the grey value after the inverse conversion of the gelatin strip. The result was compared with that of the sham operation group and the relative values were used to represent the relative activities of MMP-2 and MMP-9 for each group.

*Immune fluorescent staining of Cx43*

Immunofluorescence staining was conducted on the frozen section of the left ventricular central tissue sections of the infarcted rat hearts for 2 weeks using the method described in the literature (15). Briefly, four specimens obtained from each group were marked with anti-Cx43 murine monoclonal antibodies (ab79010; Abcam, UK). Subsequently the sections were incubated with goat anti-rat IgG combined with FITC. Afterward, the sections were observed and photographed under confocal microscopy (Zeiss LSM510 META, Germany). 

*Western blot analysis of Cx43*

Six heart samples were obtained from each group. The ventricular septa and right ventricle myocardia were cut into 1 mm sections along the grey region of the infarct and placed in liquid nitrogen for cryopreservation. The remaining myocardia were pulverized and boiled with SDS specimen buffer (0.25 M Tris–HCl, pH 6.8, 4% SDS, 40% glycerol, and 0.002% bromophenol blue) for 10 min after centrifugation. The proteins were then extracted. The equivalent protein was then purified via SDS-PAGE using 10% gel, and transferred on a nitrocellulose membrane. The membrane was blocked with 5% skimmed milk at room temperature for 2 h. Then, the proteins were incubated overnight with rabbit-derived anti-Cx43 (1:4000) and rat-derived anti-GAPDH (1:4000) at 4 °C. The samples were then incubated for 1 h with goat anti-mouse or anti-rabbit–HRP at room temperature. GADPH was used as the loading control. The density of each band was determined using the corresponding GAPDH value. 

*Data and statistics*

All numerical data are presented as mean ± S.D. The number of animals in each group was not less than 18. The differences in echocardiographic measurements, western blot analysis, and MMP zymography were compared using one-way ANOVA followed by Turdey post hoc test. The occurrence of ventricular tachycardia was compared using a chi square test. The differences with p < 0.05 were considered significant.

## Results

**Figure 1:**
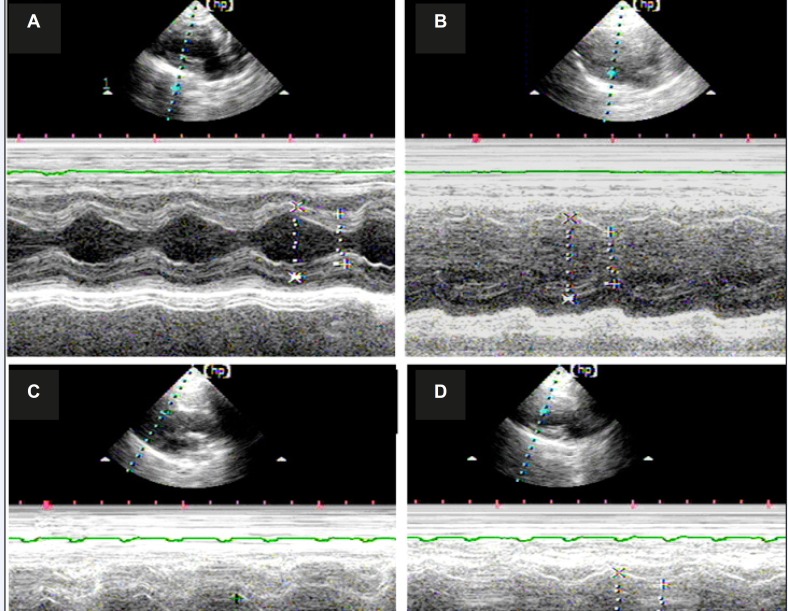
Typical M Echocardiography map of all groups shows that the heart is enlarged after myocardial infarction, whereas the ranges of relative motion of the ventricular septum and left ventricular wall were decreased. A) Echocardiogram of the rats in the sham operation group; B) echocardiogram of the rats in the vehicle-treated group; C) echocardiogram of rats in the MP group; and D) echocardiogram of the rats in the DOX group

A total of 120 rats were used in the experiment. The mortality rate after myocardial infarction in each group was about 31% and no statistical difference was observed between groups. Each group underwent echocardiography two weeks after the operation to assess the size of the left ventricle and heart function. The typical echocardiography images of all groups are shown in Figure 1 The morphology and heart function index of the left ventricle are shown in Table 1. 

**Table 1:** Heart function parameters of rats in all groups

	**Sham**	**Vehicle**	**MP**	**DOX**
Group size	27	22	20	22
HR (bpm)	311 ± 18	304 ± 15	297 ± 14	302 ± 16
LVDd (mm)	4.88 ± 0.63	6.54 ± 0.67*	6.61 ± 0.83*	5.38 ± 0.43‡§
LVSd (mm)	3.05 ± 0.55	5.01 ± 0.58*	5.20 ± 0.91*	3.75 ± 0.57‡§
FS (%)	36 ± 2.50	23 ± 4.18*	22 ± 5.65*	31 ± 6.31§
EF (%)	73 ± 9.2	47 ± 6.6*	43 ± 7.3*	56 ± 5.8*‡§

MP = methylprednisolone, DOX = doxycycline, HR = heart rate, LVSd = Left ventricular end-systolic diameter, LVEDd = Left ventricular end-diastolic diameter, FS = Left ventricular fractional shortening, EF= Left ventricular ejection fraction.

* vs. sham group p<0.05,

‡ vs. vehicle group p<0.05,

§ vs. MP group p<0.05.

No significant difference in heart rate was observed among all groups. Both the left ventricle end-diastole interior diameters (LVDd) and the left ventricle end-systole interior diameters (LVSd) increased for the rats in all MI groups compared with those in the sham operation group (p<0.05, respectively), at the same time, both the left ventricle ejection fraction (EF) and the left ventricle FS were decreased in the MI groups (p<0.05, in all groups). However, no significant difference was observed between the vehicle group and the MP group. In the DOX group, both LVDd and LVSd decreased significantly compared with the other MI groups (p<0.05, respectively). At the same time, the EF value were significantly increased in the DOX group compared with the vehicle group or MP group (p<0.05, respectively).

**Figure 2:**
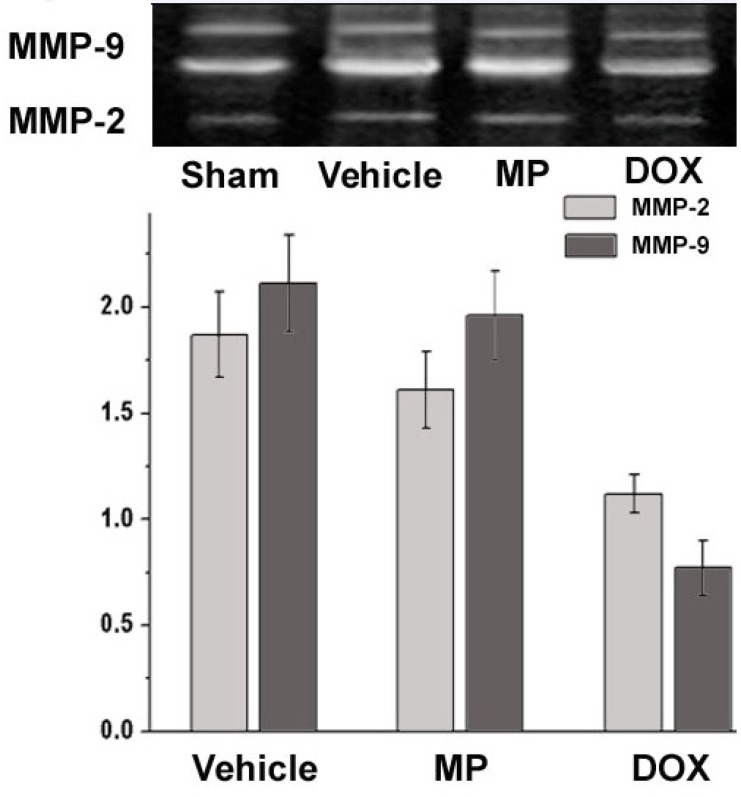
The figure above shows the gelatin zymography analysis band of the typical MMP-2 and MMP-9 in all groups. The figure below shows the relative activities of MMP-2 and MMP-9 in all groups compared with that of the sham operation group

Analysis of MMP-2 and MMP-9 activity using gelatin zymography is shown in Figure 2. MMP-2 was the translucent band near 72 kDa and MMP-9 were the two translucent bands near 94 kDa. The higher degree of translucence shows stronger activity (16).

The activity of MMP-2 and MMP-9 in the sham operation group was used as the reference value. The relative values for MMP-2 in the Vehicle, MP, and DOX groups were 1.87 ± 0.2, 1.61 ± 0.18, and 1.12 ± 0.09, and the relative values for MMP-9 were 2.11 ± 0.23, 1.96 ± 0.21, and 0.97 ± 0.13, respectively, compared with those of the sham operation group. The activities of the MMP-2 and MMP-9 in the MP treatment group after two weeks were higher than those in the vehicle treatment group. The activity of MMP-2 and MMP-9 in the DOX group were significantly lower than that in the vehicle and MP groups (P<0.05). Moreover, no significantly difference was observed when compared with the activity of MMP-2 and MMP-9 in the sham operation group (P>0.05). 

**Figure 3:**
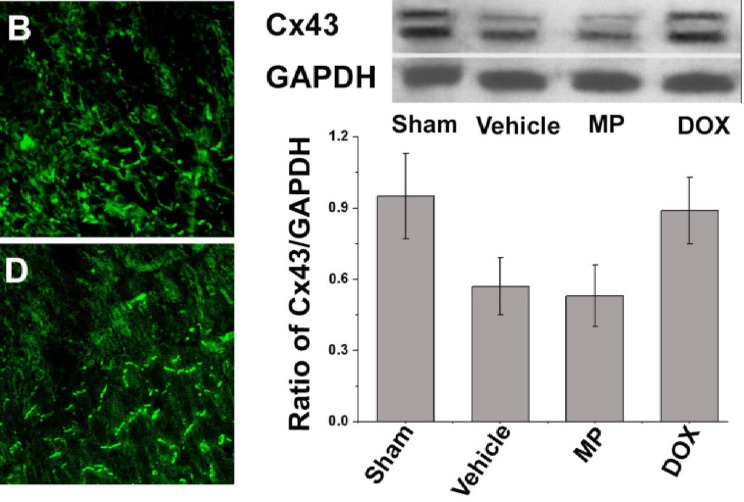
Distribution and content analysis of Cx43 in the border-zone of the infarcted area

The immunofluorescence staining indicates that Cx43 was not distributed in the infarcted area for each operation subgroup two weeks after ligation of the left anterior descending branch of the coronary artery. The distribution of Cx43 was disrupted in the border of the infarcted area and increased along the bilateral of the myocardial cells. This phenomenon was obvious in the MP group (Figure 3). The abnormal distribution of Cx43 was not observed in the septum myocardium of all groups; instead it was mainly distributed in the myocardial long axis end–end connection. In the sham operation group, whether in the anterior wall myocardium or septum myocardium, most of the Cx43 was limited to the intercalated discs of the myocardia without obvious distribution on the side edge. Similar to the immunofluorescence staining result, the relative Cx43 content in the border of the infarcted area tested under western blot analysis (0.89 ± 0.14) in the perimeter zone of the infarct in the DOX group was higher than that in the vehicle and the MP treatment groups (0.57 ± 0.12 and 0.53 ± 0.13, P>0.05，respectively). In addition, no significant difference was observed in the Cx43 content in the left ventricle anterior wall of the sham operation group (0.95 ± 0.18, P>0.05; Figure 3). 

The left Figure shows the representative image of the Cx43 distribution in the left ventricle anterior myocardium. Cx43 was marked with green fluorescence. A) Sham operation group; B) vehicle group; C) MP group; and D) DOX group. The right figure shows the western blot analysis of the relative Cx43 content in the border zone of the infarct area in all groups.

**Figure 4:**
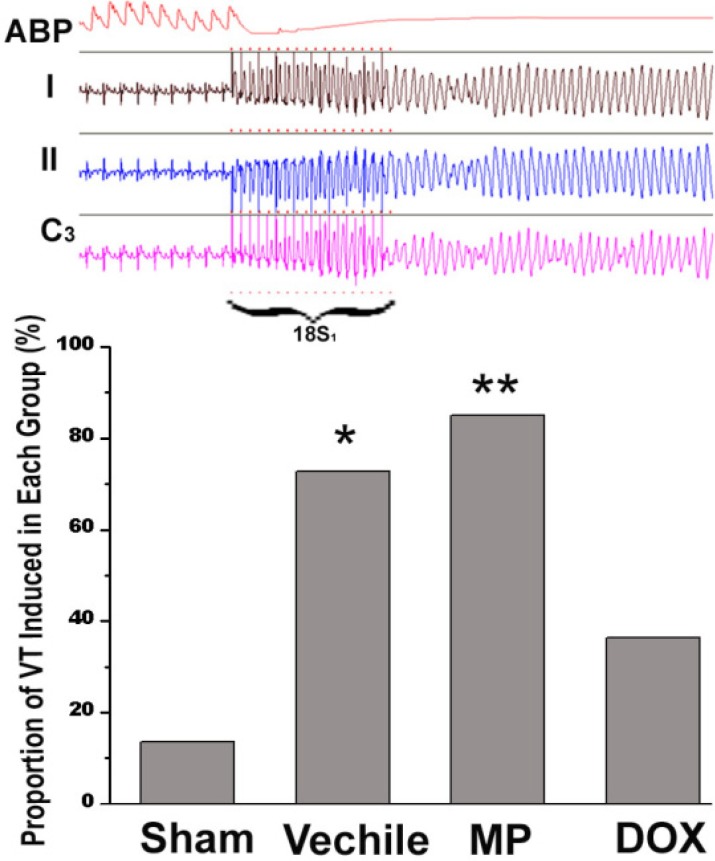
Comparison of the incidence of VT in all groups. The Figure above shows the typical VT induction curve during the convalescence stage of the rats after myocardial infarction. ABP is the aortic systolic pressure and I, II, and C3 are the electrocardiograms of the corresponding surface lead. The figure below shows the VT induction rate of each group

The electrophysiologic study results from all groups are shown in Figure 4. Epicardial stimulation induced ventricular tachycardia. Meanwhile, the curve of the blood pressure in the rat aorta showed a straight line. Ventricular tachycardia (VT) was induced in all operation groups; VT was induced only in 3/22 rats (13.6%) in the ham operation (n = 27), in 17/20 rats (85%) in the MP group (n = 20), and in 16/22 rats (72.7%) in the vehicle group (n = 22). The two groups showed significant difference compared with the sham operation group (*p *< 0.01 and p < 0.05, respectively). On the other hand, VT was induced in 8/22 rats (36.4%) in the DOX group (n = 22). The VT induction rate in the DOX group was significantly lower than those in the MP and vehicle groups (p < 0.05 and p < 0.01, respectively). The VT induction rate in the MP group was higher than that in the vehicle group; however, no statistically significant difference was observed between them. 

## Discussion

The current experiment showed that DOX effectively inhibits MMP-2 and MMP-9 activity in infarcted rats. In addition to improve the left ventricular remodeling in the infarcted rats, DOX was also prevented the decrease and redistribution of Cx43 in the border of the infarcted area. The electrophysiologic study showed that the arrhythmia induction rate for the rats treated with DOX was decreased significantly, whereas that for the rats treated with MP was not reduced. The results of our study show that the increase in MMP activity after myocardial infarction is closely related to gap junction remodeling and the occurrence of arrhythmia. Therefore, inhibiting MMP activity could improve gap junction remodeling, thereby reducing the occurrence of arrhythmia after myocardial infarction. 

Ventricular tachyarrhythmia is common in patients after MI, and is considered an important cause of sudden cardiac death. Previous studies showed that Cx43 is irregularly distributed in the myocardial tissue near the infarct, concomitant with myocardial fibrosis and changes in ion channels, and it increases the risk of ventricular arrhythmia in MI patients (17). Meanwhile, recent studies showed that a variety of MMPs increase in the myocardium after myocardial infarction, including MMP-1, MMP-2, MMP-3, MMP-7, MMP-9, MMP-11, and so on (18). Some of these enzymes are reportedly related to the occurrence of ventricular tachyarrhythmia (19). Based on MMP-7 activity, Lindsey *et al*. showed that MMPs may cause arrhythmias by influencing Cx43 (18). However, the correlation between the increased MMP activity and gap junction remodeling after myocardial infarction, and the occurrence of arrhythmia remain to be elucidated.

MMPs hydrolyze the myocardial extracellular matrix, disrupt the interconnection of myocardial cells, and inhibit the proliferation of fibrous tissue. Therefore, they play an important role in left ventricular remodeling. However, MMPs have low selectivity, thus, their hydrolytic action on myocardial extracellular proteins result in the destruction of the tight junctions in the intercalated disc. A recent study proved that MMP-2 and MMP-9 hydrolyze N-cadherin to disrupt the adhesion between cells in living tissues (20). Considering the most extensive adherence junctions in the intercalated disc are made up of N-cadherin, N-cadherin hydrolysis inevitably damages the structure of the intercalated disc between myocardial cells. 

Intercalated discs are the functional connection for signal transduction between myocardial cells and the integrity of the intercalated disc maintains the structure of gap junctions and electrical conduction. Previous studies showed that the formation and maintenance of Cx43 depends on the normal connection between myocardial cells (including adherence junctions and desmosomes). Hydrolysis of N-cadherin in the heart significantly decreases Cx43, concomitant with heart block and sudden cardiac death in model rats (4, 19). Moreover, that the knockout of vinculin, a protein that connects myocardial cells and extracellular matrix, reduces Cx43 expression and increases the incidence of ventricular tachycardia (5). The increased MMP activity after myocardial infarction hydrolyzes the myocardial extracellular matrix and destroys the connection between myocardial cells, which is an important factor in reducing Cx43 and gap junction remodeling.

To determine how the lowered MMP activity affects gap junction remodeling, DOX was introduced to inhibit MMP activity after myocardial infarction. The result showed that DOX effectively suppresses the activity of MMP-2 and MMP-9, increases the number of Cx43, and improves the redistribution of the gap junction in the border of the infarct. Our results indicate that the hydrolytic action of MMPs on the extracellular matrix causes Cx43 loss from the intercalated discs, and promotes the formation of arrhythmia substrates. In addition to gap junction remodeling, myocardial fibrosis also slows down cardiac conduction and increases susceptibility to arrhythmia after myocardial infarction. In our study, Sirius red staining was used to observe myocardial fibrosis in the perimeter zone of the infarct, and myocardial fibrosis was attenuated in the DOX-treated rat hearts**.**

In contrast to DOX, MP inhibits inflammatory reactions, but promotes MMP activity inversely. Previous studies showed that DOX improves myocardial remodeling after myocardial infarction, and MP facilitates left ventricle remodeling after infarction. Our electrophysiologic study showed that DOX reduces susceptibility to inducible ventricular tachyarrhythmia. Moreover, MP increased left ventricle myocardial remodeling in the infarcted rats and increased the incidence of inducible arrhythmias. The result of our study also show that inhibition of MMP activity after myocardial infarction lowers susceptibility to ventricular tachyarrhythmia by preventing both gap junction remodeling and left ventricular remodeling. 

In addition to gap junction remodeling, the mechanism of ventricular tachycardia after myocardial infarction is also closely related to the function of K^+^ and Ca^2+^ ion channels. The limitation of our experiment includes failure to observe the changes in the ion channels in the border of the infarct. However, we have preliminarily proven the relationship between MMPs and arrhythmia after infarction and probed into the action of MMP inhibitors in the prevention of arrhythmias and sudden death after myocardial infarction. The study provides new insight into the clinical prevention of arrhythmia after myocardial infarction. 

## Conclusion

DOX improves gap junction remodeling in the border of the infracted area while improving left ventricle remodeling and heart function, thereby reducing susceptibility to ventricular tachyarrhythmia after myocardial infarction. 

## References

[1] Zipes DP, Camm AJ, Borggrefe M, Buxton AE, Chaitman B, Fromer M, Gregoratos G, Klein G, Moss AJ, Myerburg RJ, Priori SG, Quinones MA, Roden DM, Silka MJ, Tracy C, Smith SC Jr, Jacobs AK, Adams CD, Antman EM, Anderson JL, Hunt SA, Halperin JL, Nishimura R, Ornato JP, Page RL, Riegel B, Blanc JJ, Budaj A, Dean V, Deckers JW, Despres C, Dickstein K, Lekakis J, McGregor K, Metra M, Morais J, Osterspey A, Tamargo JL, Zamorano JL (2006). guidelines for management of patients with ventricular arrhythmias and the prevention of sudden cardiac death: A report of the american college of cardiology/american heart association task force and the european society of cardiology committee for practice guidelines (writing committee to develop guidelines for management of patients with ventricular arrhythmias and the prevention of sudden cardiac death): Developed in collaboration with the european heart rhythm association and the heart rhythm society. Circulation.

[2] Akar JG, Akar FG (2007). Regulation of ion channels and arrhythmias in the ischemic heart. J. Electrocardiol.

[3] Yao JA, Hussain W, Patel P, Peters NS, Boyden PA, Wit AL (2003). Remodeling of gap junctional channel function in epicardial border zone of healing canine infarcts. Circ. Res.

[4] Li J, Levin MD, Xiong Y, Petrenko N, Patel VV, Radice GL (2008). N-cadherin haploinsufficiency affects cardiac gap junctions and arrhythmic susceptibility. J. Mol. Cell Cardiol.

[5] Zemljic-Harpf AE, Miller JC, Henderson SA, Wright AT, Manso AM, Elsherif L, Dalton ND, Thor AK, Perkins GA, McCulloch AD, Ross RS (2007). Cardiac-myocyte-specific excision of the vinculin gene disrupts cellular junctions, causing sudden death or dilated cardiomyopathy. Mol. Cell Biol.

[6] Jugdutt BI (2003). Ventricular remodeling after infarction and the extracellular collagen matrix: When is enough enough?. Circulation.

[7] Lindsey ML (2004). MMP induction and inhibition in myocardial infarction. Heart Fail. Rev.

[8] Dwivedi A, Slater SC, George SJ (2009). MMP-9 and -12 cause n-cadherin shedding and thereby beta-catenin signalling and vascular smooth muscle cell proliferation. Cardiovasc. Res.

[9] Zhu H, Wang H, Zhang X, Hou X, Cao K, Zou J (2010). Inhibiting n-cadherin-mediated adhesion affects gap junction communication in isolated rat hearts. Mol. Cells.

[10] Golub LM, Sorsa T, Lee HM, Ciancio S, Sorbi D, Ramamurthy NS, Gruber B, Salo T, Konttinen YT (1995). Doxycycline inhibits neutrophil (pmn)-type matrix metalloproteinases in human adult periodontitis gingiva. J. Clin. Periodontol.

[11] Scarabelli TM, Knight R, Stephanou A, Townsend P, Chen-Scarabelli C, Lawrence K, Gottlieb R, Latchman D, Narula J (2006). Clinical implications of apoptosis in ischemic myocardium. Curr. Probl. Cardiol.

[12] Sawicki G, Leon H, Sawicka J, Sariahmetoglu M, Schulze CJ, Scott PG, Szczesna-Cordary D, Schulz R (2005). Degradation of myosin light chain in isolated rat hearts subjected to ischemia-reperfusion injury: A new intracellular target for matrix metalloproteinase-2. Circulation.

[13] Villarreal FJ, Griffin M, Omens J, Dillmann W, Nguyen J, Covell J (2003). Early short-term treatment with doxycycline modulates postinfarction left ventricular remodeling. Circulation.

[14] Garcia RA, Go KV, Villarreal FJ (2007). Effects of timed administration of doxycycline or methylprednisolone on post-myocardial infarction inflammation and left ventricular remodeling in the rat heart. Mol. Cell Biochem..

[15] Saffitz JE, Green KG, Kraft WJ, Schechtman KB, Yamada KA (2000). Effects of diminished expression of connexin43 on gap junction number and size in ventricular myocardium. Am. J. Physiol. Heart Circ. Physiol.

[16] Hori Y, Kunihiro S, Sato S, Yoshioka K, Hara Y, Kanai K, Hoshi F, Itoh N, Higuchi S (2009). Doxycycline attenuates isoproterenol-induced myocardial fibrosis and matrix metalloproteinase activity in rats. Biol. Pharm. Bull.

[17] Nattel S, Maguy A, Le Bouter S, Yeh YH (2007). Arrhythmogenic ion-channel remodeling in the heart: Heart failure, myocardial infarction, and atrial fibrillation. Physiol. Rev.

[18] Lindsey ML, Escobar GP, Mukherjee R, Goshorn DK, Sheats NJ, Bruce JA, Mains IM, Hendrick JK, Hewett KW, Gourdie RG, Matrisian LM, Spinale FG (2006). Matrix metalloproteinase-7 affects connexin-43 levels, electrical conduction, and survival after myocardial infarction. Circulation.

[19] Li J, Patel VV, Kostetskii I, Xiong Y, Chu AF, Jacobson JT, Yu C, Morley GE, Molkentin JD, Radice GL (2005). Cardiac-specific loss of n-cadherin leads to alteration in connexins with conduction slowing and arrhythmogenesis. Circ. Res.

